# Genomic selection for crossbred performance accounting for breed-specific effects

**DOI:** 10.1186/s12711-017-0328-z

**Published:** 2017-06-26

**Authors:** Marcos S. Lopes, Henk Bovenhuis, André M. Hidalgo, Johan A. M. van Arendonk, Egbert F. Knol, John W. M. Bastiaansen

**Affiliations:** 1 Topigs Norsvin Research Center, P.O. Box 43, 6640 AA Beuningen, The Netherlands; 2 Animal Breeding and Genomics Centre, Wageningen University, 6708 PB Wageningen, The Netherlands; 3 Topigs Norsvin, Curitiba, PR 80.420-210 Brazil

## Abstract

**Background:**

Breed-specific effects are observed when the same allele of a given genetic marker has a different effect depending on its breed origin, which results in different allele substitution effects across breeds. In such a case, single-breed breeding values may not be the most accurate predictors of crossbred performance. Our aim was to estimate the contribution of alleles from each parental breed to the genetic variance of traits that are measured in crossbred offspring, and to compare the prediction accuracies of estimated direct genomic values (DGV) from a traditional genomic selection model (GS) that are trained on purebred or crossbred data, with accuracies of DGV from a model that accounts for breed-specific effects (BS), trained on purebred or crossbred data. The final dataset was composed of 924 Large White, 924 Landrace and 924 two-way cross (F1) genotyped and phenotyped animals. The traits evaluated were litter size (LS) and gestation length (GL) in pigs.

**Results:**

The genetic correlation between purebred and crossbred performance was higher than 0.88 for both LS and GL. For both traits, the additive genetic variance was larger for alleles inherited from the Large White breed compared to alleles inherited from the Landrace breed (0.74 and 0.56 for LS, and 0.42 and 0.40 for GL, respectively). The highest prediction accuracies of crossbred performance were obtained when training was done on crossbred data. For LS, prediction accuracies were the same for GS and BS DGV (0.23), while for GL, prediction accuracy for BS DGV was similar to the accuracy of GS DGV (0.53 and 0.52, respectively).

**Conclusions:**

In this study, training on crossbred data resulted in higher prediction accuracy than training on purebred data and evidence of breed-specific effects for LS and GL was demonstrated. However, when training was done on crossbred data, both GS and BS models resulted in similar prediction accuracies. In future studies, traits with a lower genetic correlation between purebred and crossbred performance should be included to further assess the value of the BS model in genomic predictions.

## Background

In pig breeding, selection takes place in purebred lines and genetic evaluations are performed mainly with information that is collected on purebreds, in high-health environments, although the final product of the pig industry is a crossbred animal. This strategy may not be optimal when the objective is to improve crossbred performance. Genetic progress realized at the purebred level may not fully translate to improved crossbred performance (under field conditions) when the genetic correlation between purebred and crossbred performance is less than 1 [1, 2]. Low genetic correlations between purebred and crossbred performance in pigs have been reported for many production traits [3, 4] and can be caused by genotype-by-environment interaction, non-additive biological (or functional) effects (such as dominance and epistasis) or breed-specific effects of (genetic marker) alleles. Therefore, if the goal is to improve crossbred performance by selection in purebreds, effects that influence the genetic correlation between purebred and crossbred performance must be evaluated. In addition, the use of crossbred data in genetic evaluations must be considered [1, 5–8].

Using either real or simulated data, several studies have investigated the relevance of genotype-by-environment interactions and dominance effects for pig breeding [9–14]. However, to date, breed-specific effects of genetic marker alleles have not been extensively studied. Breed-specific effects are observed when the same allele, say allele *A*, of a given marker has a different effect on the crossbred phenotype depending on its breed origin. Breed-specific effects at genetic markers may occur when the linkage disequilibrium (LD) between markers and quantitative trait loci (QTL) differ between breeds or when the allele frequencies of the QTL vary across breeds [6]. When breed-specific effects are present, allele substitution effects will differ between breeds, and therefore, the breeding values that are estimated by using only data from one of the purebred parental line may not accurately predict crossbred performance.

With the recent availability of high-density marker genotypes on both purebred and crossbred animals, we can now include crossbred data in genomic evaluations. Breed origin of alleles in crossbreds can also be determined and used to build breed-specific relationship matrices, as proposed by Christensen et al. [15]. Replacing the genomic relationship of a traditional genomic selection model (GS model) by the breed-specific relationship matrices (BS model), allows us to quantify the contributions of each parental breed to the additive genetic variance of the trait in crossbreds. In addition, we can also estimate breed-specific breeding values that can be backsolved for breed-specific marker effects [16]. Breed-specific marker effects could then be used to predict direct genomic values of purebred animals for crossbred performance, which would make it possible to benefit from training on crossbred field data.

In simulation studies, Ibanez-Escriche et al. [6] concluded that the BS model may not be required to effectively select purebreds for crossbred performance, while Esfandyari et al. [7] concluded that accounting for the breed origin of alleles can substantially improve accuracy of genomic prediction if the size of the training population is sufficiently large and the parental breeds are not very closely related. Applying the method proposed by Christensen et al. [15] to real pig data, Xiang et al. [17] concluded that a BS model is a good method for selecting purebreds for crossbred performance, resulting in higher prediction accuracy than the GS model. However, further studies using real data are still necessary to determine the relevance of breed-specific effects for genomic prediction. In this study, we investigated the value of breed-specific effects for predicting crossbred performance using real data. First, the contribution of each parental breed to the genetic variance was quantified for traits that were measured in a two-way crossbred population. Second, prediction accuracies were estimated with the GS and BS model, using either purebred or crossbred training data.

## Methods

### Ethics statement

The data used for this study were obtained as part of routine data recording in a commercial breeding program. Samples collected for DNA extraction were only used for routine diagnostic purposes of the breeding program. Data recording and sample collection were conducted strictly in line with the rules given by Dutch Animal Research Authorities.

### Data

Phenotypic and genotypic data were available for pigs from two purebred populations: Large White (LW) and Landrace (LR), and from a two-way crossbred population (F1) that consisted of animals produced by reciprocal crosses of the purebred populations (LW♂ × LR♀ and LR♂ × LW♀). Phenotypic data were available for litter size (LS, sum of piglets born alive and stillborn in the same litter) and gestation length (GL, number of days between insemination and farrowing). Both traits were recorded from parities 2 to 7. Records from the first parity were excluded because LS and GL measured in the first versus later parities have been described as different traits based on low genetic correlation [18, 19].

Phenotypic data on both traits were available for 22,597 LW, 27,035 LR, and 29,847 F1 animals (Table 1). The F1 population consisted of 14,964 animals from the LW♂ × LR♀ cross, and 14,883 animals from the LR♂ × LW♀ cross. On average, data from 3.8, 3.6, and 2.5 parities per animal were available in the LW, LR, and F1 populations, respectively. Data from the purebred populations were recorded on genotyped animals (3723 LW and 3291 LR) and their non-genotyped contemporaries (i.e. animals from the same breed and farm as the genotyped animals; 18,874 LW and 23,744 LR). The purebred animals were located on 18 (LW) and 20 (LR) farms and were born between 2004 and 2014. Data from the F1 population were recorded on 1126 genotyped animals and their 1120 non-genotyped contemporaries. These genotyped F1 animals and their contemporaries were located on six farms. Finally, data were also recorded on 27,601 non-genotyped F1 offspring of the genotyped purebred animals. This additional group of F1 animals was located across 111 farms and was only used to increase the size of the crossbred population to estimate the genetic correlation between purebred and crossbred performance. All F1 animals were born between 2010 and 2014.

**Table 1:** Summary statistics

Population^a^	Phenotypes^b^		Genotypes^c^	Genotypes and phenotypes^d^		Mean ± standard deviation^e^	
	Animals	Records	Animals	Animals	Records	LS	GL
LW	22,597	84,837	3723	924	3358	15.91 ± 3.71	115.38 ± 1.63
LR	27,035	96,431	3291	924	3319	15.40 ± 3.56	116.10 ± 1.61
F1	29,847	75,143	1126	924	3771	15.93 ± 3.59	115.12 ± 1.49

^a^Large White (LW), Landrace (LR), and two-way crossbred (F1)

^b^Number of animals with phenotypic information and total number of phenotypic records for these animals

^c^Number of genotyped animals used for imputation and phasing procedures

^d^Number of genotyped animals and number of phenotypic records for these animal used for estimating the variance components and SNP effects

^e^Mean ± standard deviation of litter size (LS) and gestation length (GL) of the populations used for estimating the variance components and SNP effects

Phenotypes of genotyped animals were pre-adjusted for fixed effects using the larger dataset, i.e. including contemporaries, such that fixed effects were accounted for more accurately. Fixed effects were estimated by fitting a single-trait, pedigree-based mixed linear model for each population, using ASReml v3.0 [20]. The model used for LS included the fixed effects of parity, interval between weaning and pregnancy (days), whether more than one insemination was performed or not (yes or no), litter type (whether the boar used for the inseminations was from the same breed as the sow, i.e. purebred litter, or from a different breed i.e. crossbred litter), and herd-year-season, and the random effects of service sire, permanent environmental effects, and additive genetic effects. The model used for GL included the fixed effects of parity, whether more than one insemination procedure was performed or not (yes or no), litter type (purebred or crossbred), herd-year-season, and the covariate LS. The random effects of the model for GL were the same as for LS. When evaluating the performance of the F1 animals, the fixed effect of litter type was not included in the model for either trait.

### Genetic correlations between purebred and crossbred performance

Genetic correlations between purebred and crossbred performance for both traits were estimated using a three-trait model, as described by Lutaaya et al. [21]. Performance in each population (two purebreds and one crossbred) was considered as a different trait. The three-trait model was implemented in ASReml 3.0 [20]. Effects accounted for in the three-trait model were the same as those accounted for in the single-trait model that was used for the pre-adjustment of the phenotypes, except that a fixed effect for breed reciprocity was added for crossbred performance (LR♂ × LW♀ or LW♂ × LR♀).

### Genotyping

Genotyping was performed mainly using the Illumina Porcine SNP60 Beadchip, but some animals from all populations were genotyped using the Illumina Porcine SNP60 v2 Beadchip. Genotypic data were available on 3723 LW, 3291 LR, and 1126 F1 animals (Table 1). In the purebred populations, both males and females were genotyped. In the F1 population, only females were genotyped. Genotypes of all animals were imputed to the SNP60 Beadchip for all SNPs that passed the quality control. The quality control excluded SNPs with a GenCall lower than 0.15, a call rate lower than 0.95, a minor allele frequency lower than 0.01, and SNPs that deviated significantly from Hardy–Weinberg equilibrium (χ^2^ > 600). SNPs located on the sex chromosomes and unmapped SNPs were also excluded. Positions of the SNPs were based on the Sscrofa10.2 assembly of the reference genome [22]. All genotyped animals had a frequency of missing genotypes below the threshold of 0.05 in order to exclude poorly genotyped animals. After quality control and imputation, 39,788 SNPs for LW, 41,299 SNPs for LR, and 45,515 SNPs for F1 were available for further analyses.

### Imputation and phasing of the genotype data

Imputation and phasing of the genotype data were performed using AlphaImpute [23], combining genomic and pedigree information to determine the parental origin of alleles. Imputation of missing genotypes of the purebred populations was performed within populations using all SNPs that passed quality control. For the F1 population, imputation of missing genotypes and phasing of the data were performed by combining the F1 data with the imputed purebred data but using only the 36,733 SNPs that segregated (minor allele frequency >0.01) in each population.

To ensure the use of accurately phased haplotypes for determining breed origin of alleles, a threshold was applied to the F1 phased data. For each SNP genotype of each individual, AlphaImpute [23] generates two probabilities: P_1_ is the probability that a specific allele was inherited from the father, e.g. allele *G* of a *G*/*C* genotype, and P_2_ is the probability that the same allele was inherited from the mother. For a heterozygous animal (*CG*), for which allele *C* was inherited with certainty from the father (and therefore allele *G* from the mother), the probabilities would be P_1_ = 0 and P_2_ = 1. When the phasing cannot be performed with certainty, these probabilities will have values between 0 and 1. Values of P_1_ or P_2_ between 0.1 and 0.9 were considered to have poor phasing. SNPs that were considered poorly phased in more than 95% of animals were excluded from the dataset. Then, animals that had more than 5% poorly phased genotypes were excluded. After this quality control, 924 F1 animals with genotypes for 31,930 SNPs were available to estimate variance components and SNP effects. The same set of SNPs was also used to estimate variance components and SNP effects for the purebred populations.

After phasing of the genotype data, the breed origin of alleles was easily determined because the breeds of the parents of the F1 individuals were known. The final F1 population included 414 animals from the LW♂ × LR♀ cross and 510 animals from the LR♂ × LW♀ cross.

### Estimation of variance components and SNP effects

The number of animals with both phenotypes and genotypes was larger in the purebred than in the crossbred population. Because the size of the training population influences the estimation of SNP effects and consequently prediction accuracy [24], we randomly selected 924 animals born between 2010 and 2014 from each purebred population for use in estimation of variance components and SNP effects. Our aim was to conduct a fair comparison, since the size of the training population and range of birth years were the same for each population (Table 1). In order to have independent datasets for validation analyses (discussed below), the purebred animals used as training population had no offspring or sibs in the F1 population.

Variance components and SNP effects were estimated within each population using a traditional genomic selection model (GS model) and a model that accounts for breed-specific effects (BS model). The GS model was applied to both purebred and crossbred data, while the BS model was applied only to the crossbred data. These models were implemented in ASReml [20], as follows:

$${\mathbf{y}} = \mathbf{1}\upmu + {\mathbf{Ss}} + {\mathbf{Pp}} + {\mathbf{Zu}}_{GS} + {\mathbf{e}}\quad ({\text{GS}}\;{\text{model}})$$

$${\mathbf{y}} = \mathbf{1}\upmu + {\mathbf{Ss}} + {\mathbf{Pp}} + {\mathbf{Z}}_{LW} {\mathbf{u}}_{BS|LW}\quad + {\mathbf{Z}}_{LR} {\mathbf{u}}_{BS|LR} + {\mathbf{e}}\quad ({\text{BS}}\;{\text{model}})$$

where **y** is a vector of phenotypes pre-adjusted for fixed effects; µ is the mean of the populations and **1** a vector of 1s; **S** is the design matrix for service sire effects; **s** is an unknown vector of service sire effects; **P** is the design matrix for permanent environmental effects; **p** is an unknown vector of permanent environmental effects; **Z**, **Z**_*LW*_ and **Z**_*LR*_ are design matrices for the additive genetic effects; **u**_*GS*_ is an unknown vector of additive genetic effects (i.e. breeding values); **u**_*BS|LW*_ and **u**_*BS|LR*_ are unknown vectors of breed-specific additive genetic effects (i.e. breed-specific breeding values). Assumed distributions were ${\mathbf{s}} \sim N\left( {\mathbf{0},{\mathbf{I}}\sigma_{s}^{2} } \right)$, ${\mathbf{p}} \sim N\left( {\mathbf{0},{\mathbf{I}}\sigma_{r}^{2} } \right)$, ${\mathbf{u}}_{GS} \sim N\left( {\mathbf{0},{\mathbf{G}}\sigma_{{a_{GS} }}^{2} } \right)$, ${\mathbf{u}}_{{BS|F1_{LW} }} \sim N\left( {\mathbf{0},{\mathbf{B}}_{LW} \sigma_{{a_{{BS|F1_{LW} }} }}^{2} } \right)$, and ${\mathbf{u}}_{{BS|F1_{LR} }} \sim N\left( {\mathbf{0},{\mathbf{B}}_{LR} \sigma_{{a_{{BS|F1_{LR} }} }}^{2} } \right)$, where **I** is an identity matrix, $\sigma_{\text{s}}^{2}$ is the service sire variance, $\sigma_{r}^{2}$ is the permanent environmental variance, **G** is the traditional genomic additive relationship matrix, $\sigma_{{a_{GS} }}^{2}$ is the additive genetic variance, **B**_*LW*_ and **B**_*LR*_ are breed-specific genomic relationship matrices, and $\sigma_{{a_{{BS|F1_{LW} }} }}^{2}$ and $\sigma_{{a_{{BS|F1_{LR} }} }}^{2}$ are breed-specific additive genetic variances. Heritability was defined as $\sigma_{{a_{GS} }}^{2} /\sigma_{\text{P}}^{2}$ for the GS model and as $(\sigma_{{a_{{BS|F1_{LW} }} }}^{2} + \sigma_{{a_{{BS|F1_{LR} }} }}^{2} )/\sigma_{\text{P}}^{2}$ for the BS model, where $\sigma_{\text{P}}^{2}$ is the total phenotypic variance (sum of all variances from each model). The **G** matrix was built according to VanRaden [25]:

$${\mathbf{G}} = \frac{{{\mathbf{MM}}'}}{{2\mathop \sum \nolimits_{i = 1}^{n} p_{i} q_{i} }},$$

where *p*_*i*_ and *q*_*i*_ are the allele frequencies of the *i*th genetic marker, and **M** is a matrix of centered genotype codes (0 − 2*p*_*i*_, 1 − 2*p*_*i*_, 2 − 2*p*_*i*_). The **B**_*LR*_ and **B**_*LW*_ matrices were built according to the genomic gametic relationship matrices described by Christensen et al. [15] and Nishio and Satoh [26]:

$${\mathbf{B}} = \frac{{{\mathbf{LL}}^{\prime } }}{{\mathop \sum \nolimits_{i = 1}^{n} p_{i}^{*} q_{i}^{*} }},$$

where *p*_i_* and *q*_i_* are the frequencies of the allele codes from either *LW* (**B**_*LW*_) or *LR* (**B**_*LR*_) in the F1 population, and **L** is a matrix of centered allele codes (0 − *p*_i_*, 1 − *p*_i_*) from either breed *LW* (**B**_*LW*_) or *LR* (**B**_*LR*_). After obtaining the estimated breeding values (EBV) from the GS model (${\hat{\mathbf{u}}}_{GS}$) and the BS model (${\hat{\mathbf{u}}}_{{BS|F1_{LW} }}$ and ${\hat{\mathbf{u}}}_{{BS|F1_{LR} }}$), we backsolved these EBV to obtain estimates of SNP effects, which were used to estimate the direct genomic values (DGV) of the validation animals. Backsolving of EBV from the GS models to obtain SNP effect estimates ($\hat{\varvec{a}}_{GS}$) was performed as described by Wang et al. [16]:

$$\hat{\varvec{a}}_{GS} = \frac{{{\mathbf{M}}^{\prime } {\mathbf{G}}^{ - 1} \hat{\varvec{u}}_{GS} }}{{2\mathop \sum \nolimits_{i = 1}^{n} p_{i} q_{i} }},$$

and backsolving of EBV from the BS model to obtain breed-specific SNP effect estimates ($\hat{\varvec{a}}_{{BS|F1_{LW} }}$ and $\hat{\varvec{a}}_{{BS|F1_{LR} }}$, for LW and LR breed, respectively), was performed by extending the method described by Wang et al. [16]:

$$\hat{\varvec{a}}_{BS} = \frac{{{\mathbf{L}}^{\prime } {\mathbf{B}}^{ - 1} \hat{\varvec{u}}_{BS} }}{{\mathop \sum \nolimits_{i = 1}^{n} p_{i}^{ *} q_{i}^{ *} }}.$$

### Predicting crossbred performance

**Fig. 1:**
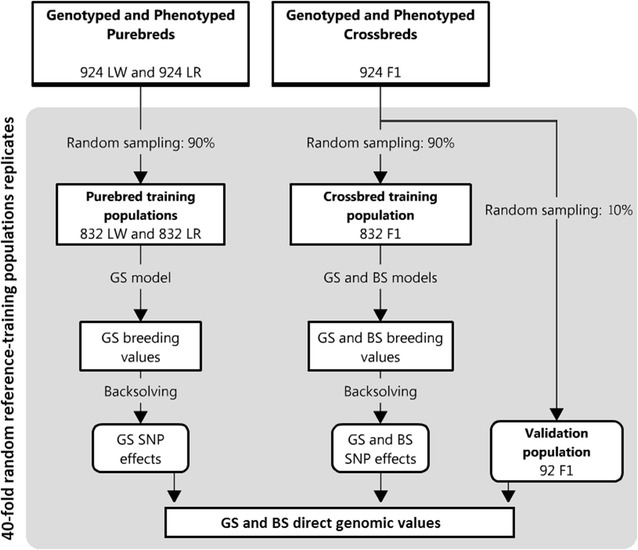
Schematic representation of the steps involved in the prediction analyses. *LW* Large-White, *LR* landrace population, *F1* two-way crossbred, *GS model* traditional genomic selection model, *BS model* model that accounts for breed-specific effects

A schematic representation of the steps involved in the prediction analyses is in Fig. 1. Individual performance of genotyped crossbred sows was predicted with the SNP effects estimated by the GS model and the BS model. A validation using a 40-fold random training-validation populations was performed to evaluate prediction accuracies. For each replicate, 10% of the genotyped F1 animals (N = 92) were randomly assigned to the validation population and the other 90% (N = 832) were assigned to the F1 training population. For the purebred training populations, 90% (N = 832) of the animals that were used to estimate variance components were randomly assigned to the training population in each replicate. Within each replicate, traditional (GS) DGV of validation animals were estimated as: $\hat{\varvec{u}}_{GS|j} = {\mathbf{M}}_{F1} \hat{\varvec{a}}_{GS|j}$, where **M**_*F*1_ is a matrix of centered genotypes of the F1 validation animals and $\hat{\varvec{a}}_{GS|j}$ is a vector of SNP effects estimated using the GS model on the training animals, where subscript *j* indicates the breed of the animals included in the training population (LW, LR or F1).

In addition, within each replicate, two types of breed-specific (BS) DGV of the validation animals were estimated. The first type of BS DGV used SNP effects estimated within the parental purebred populations as: $\hat{\varvec{u}}_{BS|j} = {\mathbf{L}}_{{F1_{j} }} \hat{\varvec{a}}_{GS|j}$, where ${\mathbf{L}}_{{F1_{j} }}$ is a matrix of centered allele codes that the F1 validation animals inherited from the *j*th parental purebred populations (LW or LR) and $\hat{\varvec{a}}_{GS|j}$ is defined as above. Thus, separate BS DGV were estimated, one for each parental purebred population. In addition, total the BS DGV ($\hat{\varvec{u}}_{BS|LW} + \hat{\varvec{u}}_{BS|LR}$) was calculated for the validation animals. The second type of BS DGV used SNP effects that were estimated within the crossbred population as: $\hat{\varvec{u}}_{{BS|F1_{j} }} = {\mathbf{L}}_{{F1_{j} }} \hat{\varvec{a}}_{{BS|F1_{j} }}$, where $\hat{\varvec{a}}_{BS|F1j}$ is a vector of SNP effects that were estimated using the BS model on the alleles that the F1 training animals inherited from the *j*th parental purebred populations, and ${\mathbf{L}}_{{F1_{j} }}$ is defined as above. Total BS DGV ($\hat{\varvec{u}}_{{BS|F1_{LW} }} + \hat{\varvec{u}}_{{BS|F1_{LR} }}$) were also calculated for the validation animals.

Prediction accuracy was defined as the correlation of the GS DGV, the BS DGV, or the total BS DGV with pre-adjusted phenotypes in the validation population. Prediction accuracies presented are averages over 40-fold random reference-training population replicates.

## Results

The estimate of the pedigree-based genetic correlation between purebred LW and crossbred performance was 0.91 ± 0.04 for LS and 0.92 ± 0.02 for GL (Table 2). The estimate of the pedigree-based genetic correlation between purebred LR and crossbred performance was slightly lower, i.e. 0.89 ± 0.04 for LS and 0.88 ± 0.03 for GL. Estimates of the pedigree-based heritability for all traits and populations are provided in Table 2.

**Table 2:** Estimates of pedigree-based heritability (*h* ^2^) and genetic correlation between purebred and crossbred (*r*_*pc*_) populations from a three-trait model

Population^a^	*h* ^2^	*r*_*pc*_
Litter size		
LW	0.18 ± 0.01	0.91 ± 0.04
LR	0.14 ± 0.01	0.89 ± 0.04
F1	0.14 ± 0.01	
Gestation length		
LW	0.39 ± 0.01	0.92 ± 0.02
LR	0.39 ± 0.01	0.88 ± 0.03
F1	0.37 ± 0.01	

^a^Populations used in the analyses were Large White (LW), Landrace (LR), and two-way crossbred (F1)

Using the GS model, estimates of the heritability of 0.15 ± 0.03 and 0.12 ± 0.03 were obtained for LS in the LW and LR populations, respectively (Table 3). Using the GS and BS models, estimates of the heritability for LS in the F1 population were similar (0.12 ± 0.03). Using the GS model, estimates of the heritability of 0.34 ± 0.04 and 0.33 ± 0.04 were obtained for GL in the LW and LR populations, respectively (Table 3). For GL in the F1 population, estimates of the heritability of 0.39 ± 0.04 and 0.40 ± 0.04 were obtained with the GS and BS models, respectively. For both traits, the estimate of the breed-specific additive genetic variance was slightly larger for alleles that were inherited from the LW population compared to alleles that were inherited from the LR population, although the standard errors were high (0.74 ± 0.23 and 0.56 ± 0.24 for LS, and 0.42 ± 0.08 and 0.40 ± 0.08 for GL, respectively).

**Table 3:** Estimates of variance components and (±) standard errors for litter size and gestation length

Population^a^	Model^b^	$\sigma_{s}^{2}$	$\sigma_{r}^{2}$	$\sigma_{a}^{2}$	$\sigma_{{a_{LW} }}^{2}$	$\sigma_{{a_{LR} }}^{2}$	$\sigma_{e}^{2}$	*h* ^2^
Litter size								
LW	GS	0.43 ± 0.13	1.70 ± 0.30	1.81 ± 0.36	–	–	8.26 ± 0.25	0.15 ± 0.03
LR	GS	0.02 ± 0.09	1.22 ± 0.28	1.30 ± 0.31	–	–	8.80 ± 0.27	0.12 ± 0.03
F1	GS	0.10 ± 0.10	1.38 ± 0.28	1.42 ± 0.34	–	–	8.54 ± 0.24	0.12 ± 0.03
F1	BS	0.11 ± 0.10	1.37 ± 0.29	–	0.74 ± 0.23	0.56 ± 0.245	8.52 ± 0.24	0.12 ± 0.03
Gestation length								
LW	GS	0.21 ± 0.03	0.33 ± 0.05	0.68 ± 0.09	–	–	0.78 ± 0.02	0.34 ± 0.04
LR	GS	0.23 ± 0.03	0.26 ± 0.05	0.64 ± 0.09	–	–	0.82 ± 0.03	0.33 ± 0.04
F1	GS	0.16 ± 0.02	0.23 ± 0.06	0.81 ± 0.11	–	–	0.90 ± 0.03	0.39 ± 0.04
F1	BS	0.16 ± 0.02	0.17 ± 0.06	–	0.42 ± 0.08	0.40 ± 0.08	0.90 ± 0.03	0.40 ± 0.04

Variance components: service sire ($\sigma_{s}^{2}$), permanent environment ($\sigma_{r}^{2}$), additive ($\sigma_{a}^{2}$), additive for the alleles of the F1 population inherited from the LW ($\sigma_{{a_{LW} }}^{2}$) and LR ($\sigma_{{a_{LR} }}^{2}$) populations, and error ($\sigma_{e}^{2}$). *h* ^2^: heritability

^a^Large White (LW), Landrace (LR), two-way crossbred (F1)

^b^Traditional genomic selection model (GS) and a model that accounts for breed-specific effects (BS)

The highest accuracy for predicting the performance of crossbred sows was observed when training was done on crossbred data (Table 4), for which the GS and BS models resulted in similar prediction accuracies. For LS, when training was done on crossbred data, prediction accuracy was the same for the GS DGV and the total BS DGV (0.23 ± 0.08). For GL, when training was done on crossbred data, similar prediction accuracies were obtained for the total BS DGV and the GS DGV (0.53 ± 0.08 and 0.52 ± 0.08, respectively). For both traits, the BS DGV based on the LW alleles resulted in higher prediction accuracies than the BS DGV based on the LR alleles (0.21 ± 0.08 vs. 0.12 ± 0.09 for LS; 0.43 ± 0.08 vs. 0.34 ± 0.09 for GL).

**Table 4:** Prediction accuracy of performance of crossbred sows for gestation length and litter size

Model^a^	Training^b^	Accuracy^c^	SD^d^
Litter size			
GS	LW	0.06	0.10
	LR	0.07	0.11
	F1	**0.23**	0.08
BS	LW	0.06	0.11
	LR	0.06	0.13
	LW and LR*	0.09	0.12
	F1_LW_	0.21	0.08
	F1_LR_	0.12	0.09
	F1_LW_ and F1_LR_*	**0.23**	0.08
Gestation length			
GS	LW	0.42	0.08
	LR	0.30	0.09
	F1	**0.52**	0.08
BS	LW	0.39	0.08
	LR	0.23	0.10
	LW and LR*	0.45	0.08
	F1_LW_	0.43	0.08
	F1_LR_	0.34	0.09
	F1_LW_ and F1_LR_*	**0.53**	0.08

* Predicted direct genomic value was the “total direct genomic value” (sum of the breed-specific direct genomic values)

^a^GS, traditional genomic selection model; BS, model that accounts for breed-specific effects

^b^LW, Large White; LR, Landrace; F1, two-way crossbred; F1_LW_, alleles of the F1 population inherited from the LW population; F1_LR_, alleles of the F1 population inherited from the LR population. Training populations in each replicate were defined as a random set of 90% (N = 832) of the animals used for the estimation of variance components

^c^Average of the 40 replicates; accuracy was defined as the correlation between the direct genomic values of the validation population [random set of 10% (N = 92) of the crossbred animals used for the estimation of variance components] and their average pre-adjusted phenotypes in each replicate

^d^Standard deviation over replicates; the highest accuracies for each model and trait are indicated in bold

## Discussion

In this study, we showed, that, for LS and GL, the same SNP allele in the F1 population can contribute differently to the additive genetic variance depending on its breed origin (Table 3) and that accounting for this difference only has a small impact on prediction accuracy (Table 4). The standard errors of the breed-specific variance estimates were rather high, as expected with a small dataset (N = 924), especially for LS, for which it reached 43% of the variance estimate (Table 3) and, thus, these results must be considered carefully. While standard errors can increase due to inaccurate determination of the breed origin of alleles, this increase is expected to be limited because a stringent quality control was applied to the phased genotypes.

Predicting performance of genotyped crossbred sows was more accurate when training was on crossbred data instead of purebred data (Table 4). The superiority of training on crossbred data compared to purebred data for predicting crossbred performance is in line with previous studies that were carried out using both simulated and real data. In simulation studies, training on crossbred data has been reported to yield either similar [27] or higher accuracies [7, 17] compared to training on purebred data, while using real data, training on crossbred data has been found to yield the highest prediction accuracies [2, 28].

The GS and BS models resulted in similar prediction accuracies when training was on crossbred data. Greater benefits of using the BS model over the GS model are expected when crossbred populations are larger and more distant parental breeds are crossed [7]. In the current study, we evaluated a small F1 population (N = 924) that was obtained by crossing two dam lines. If a sire line was used instead as one of the parental line, the parental breeds may, depending on the lines chosen, be more distant [29] and the BS model could have a larger impact on prediction accuracy. In pig breeding, the cross between sire and dam lines is typically done by mating F1 sows to boars from a sire line in the next generation. Therefore, we expect that applying the BS model to such a three-way crossbred population may result in larger benefits over the GS model. However, to evaluate three-way crossbred populations, even larger crossbred populations may be required because each crossbred will only carry two (grand) parental alleles.

Predictions of the performance of genotyped crossbred sows using BS DGV based on alleles of the LW breed resulted in higher accuracies than using BS DGV from alleles of the LR breed. This advantage of the LW breed compared to the LR breed when training was on crossbred data is consistent with the larger amount of variance that is explained by alleles of the LW breed (Table 3) and also with the higher estimate of the pedigree-based genetic correlation between the LW and F1 populations compared to that between the LR and F1 populations (Table 2). Furthermore, when training was on purebred data, the performance of genotyped crossbred sows was more accurately estimated when total BS DGV were used than when GS DGV based on SNP effects estimated in each purebred were used (Table 4). This suggests that determining the breed origin of the alleles in crossbred sows is beneficial, even if the training is on purebred data.

In this study, the BS model was applied to a training population composed of crossbred animals only. As a further step, the benefits of accounting for breed-specific effects could be evaluated under combined crossbred and purebred selection (CCPS), which has been described as an efficient way of increasing genetic progress in both purebred and crossbred populations [5, 30]. Such an approach was proposed by Christensen et al. [15] and further evaluated by Christensen et al. [31] and consists of performing genomic evaluation using a combination of the genomic relationship of the purebred populations and the breed-specific relationship matrices of the crossbred population. One of the limitations of applying CCPS using pedigree-based models is that it also resulted in increased rates of inbreeding [32]. However, with genomic-based models this increased inbreeding from CCPS is expected to be limited, or even absent, because the genomic information allows estimation of Mendelian sampling and therefore reduces the emphasis on family information in selection [1].

A CCPS approach to estimate BS DGV could be applied for both two-way and three-way crossbred populations. In the current study, we evaluated a two-way crossbred population and determination of the breed origin of alleles depended on pedigree information. In three-way crossbred populations, pedigree information is not commonly recorded, and thus, different strategies would be required to determine the breed origin of alleles. Recently, Bastiaansen et al. [33] proposed a method to determine breed origin of alleles in crossbreds using long-range phasing that can be applied to crossbred populations where pedigree information is lacking and this has been further evaluated by Sevillano et al. [34] and Vandenplas et al. [35]. With this method, close relationships between the crossbred and purebred genotyped animals would not be required because long-range phasing will work even with distant purebred relatives of the crossbreds. Therefore, future studies on practical applications of BS models in CCPS should evaluate a combination of the methods proposed by Christensen et al. [15] and Bastiaansen et al. [33]. When crossbred genotypes are not available, an alternative strategy would be to apply models that account for purebred genotypes and crossbred phenotypes only, as proposed by Tusell et al. [36]. These authors showed that such a strategy improves the theoretical accuracy of selection for crossbred performance without crossbred genotypes. However, we must keep in mind that with the fast developments of genotyping platforms and techniques, the major cost limitation for the use of crossbred data in genomic evaluation may come from obtaining phenotypes rather than genotypes.

In this study, we investigated the relevance of breed-specific effects when genomic selection is applied to real data for two reproductive traits in pigs. In future studies, traits that have a lower genetic correlation between purebred and crossbred performance should be included because benefits of BS models are expected to be larger in those cases. In addition to studying less correlated traits, investigating breed-specific effects in crosses of more distant purebred populations may result in larger benefits of the BS model. Furthermore, evaluation of larger datasets than those used in the current study is also required for more conclusive results and to quantify the benefits of accounting for breed-specific effects in prediction models.

## Conclusions

In this study, we provide evidence of breed-specific SNP effects for litter size and gestation length in a two-way crossbred population. Predicting performance of crossbred sows was shown to be more accurate when training was performed on crossbred instead of purebred data. However, when training was done on crossbred data, the GS and BS models resulted in similar prediction accuracies. In future studies, traits with lower genetic correlations between purebred and crossbred performance should be evaluated to confirm the potential benefit of BS models in genomic predictions.
